# Quality and Reliability Analysis of YouTube Videos Related to Neonatal Sepsis

**DOI:** 10.7759/cureus.38422

**Published:** 2023-05-02

**Authors:** Handan Hakyemez Toptan, Ali Kizildemir

**Affiliations:** 1 Neonatology, Marmara University Pendik Training and Research Hospital, Istanbul, TUR; 2 Pediatrics, Private Medifema Hospital, Izmir, TUR

**Keywords:** quality, reliability, gqs, discern, youtube, neonatal sepsis

## Abstract

Aim

Neonatal sepsis is a clinical syndrome of illness accompanied by bacteremia that develops in the first month of life. The objective of this study was to evaluate the reliability and quality of YouTube^TM^ (www.youtube.com) videos pertaining neonatal sepsis.

Methods

The first 100 videos on YouTube^TM^ pertaining to neonatal sepsis were included in the analysis. Features like videos’ image type, content and qualification of video creators were recorded. In addition, videos’ length, upload date, time since upload, comment and like counts were also recorded. Quality of the videos was measured by the researchers using the Global Quality Scale (GQS) and reliability of the videos was evaluated through the DISCERN (Quality Criteria for Consumer Health Information on Treatment Choices) tool.

Results

The total length of the examined 100 videos was 35.84 hours and the total view count was 1,173,247. The most common video content was general information about neonatal sepsis, education and diagnosis. When qualification of the video creators was examined, the most common creators were physicians followed by other persons and health channels. The videos were divided into two groups according to the creators. Accordingly, 40 (40%) videos were uploaded by professionals and 60 by non-professionals. There was a statistically significant difference between physicians and non-physicians in terms of the mean DISCERN and GQS scores (both, p<0.01).

Conclusion

Both DISCERN and GQS scores were statistically significantly higher in the videos provided by physicians. Physicians should be encouraged to upload accurate informative videos about neonatal sepsis and direct parents to accurate sources of treatment.

## Introduction

Neonatal sepsis is a clinical syndrome of illness accompanied by bacteremia that develops in the first month of life and is a cause of substantial morbidity and mortality [1]. The signs and symptoms of neonatal sepsis are affected by the virulence of pathogens, the susceptibility of the host and the portal of entry [2]. The clinical manifestations range from subclinical infection to severe focal or systemic disease [3]. In USA, the incidence of neonatal sepsis varies from one to four infections per 1000 live births [4]. In the treatment of sepsis, once the pathogen is identified, the most appropriate antimicrobial(s) are administered. It is of paramount importance to raise public awareness of neonatal sepsis and provide parents with accurate and reliable information.

The Internet has become the most widely used online source of health-related information and also an educational tool for medical practitioners. Today, nearly five billion people are active Internet users worldwide [5]. People search the Internet to find out a remedy for their diseases, share experience with each other or even buy treatments [5]. Internet is the third trustworthy source of information related to healthcare after physicians and healthcare centers [6]. In the disease process, Internet can be the easily accessible tool to get information about disease management, diagnosis and medical treatment. It has been reported that 75% of patients especially those with chronic diseases learn information specific to their diseases from the Internet. However, 80% of Internet users who obtain health-related information do not share what they have learned with their physicians [7].

YouTube^TM^ is the world’s most popular video-sharing platform and has become a source of open-access information for patients to learn about their diseases [8]. Technically, YouTube^TM^ is the second largest search engine following Google. In 2022, over 2.6 billion people used YouTube^TM^ once a month worldwide. One billion YouTube^TM^ videos are watched daily and 500 hours of videos are uploaded each minute. YouTube^TM^ videos are ranked by various criteria such as view counts, comments and likes; however, these do not reflect the quality of a video. While YouTube^TM^ can provide an opportunity for healthcare seekers and also be an educational tool for medical practitioners, there are concerns about the quality and reliability of YouTube^TM^ videos because anyone can provide videos on any topic for free of charge and spread it to millions of people in a short timespan. This has prompted researchers to conduct YouTube^TM ^video analysis studies in almost every field of medicine [9-11]. However, the number of studies on YouTube^TM^ videos related to neonatology is quite limited [12]. In this study, we aimed to investigate the quality and reliability of YouTube^TM^ videos on neonatal sepsis.

## Materials and methods

This YouTube^TM^ analysis study was conducted by two experienced researchers between August 08, 2022 and August 10, 2022. Ethical approval was not deemed necessary since the study did not involve animals or humans. In addition, no permission was received from YouTube^TM^ since videos on this platform are publicly available. The evaluation was made by the two observers at the same time, and in different locations in order to avoid potential bias.

**Figure 1:**
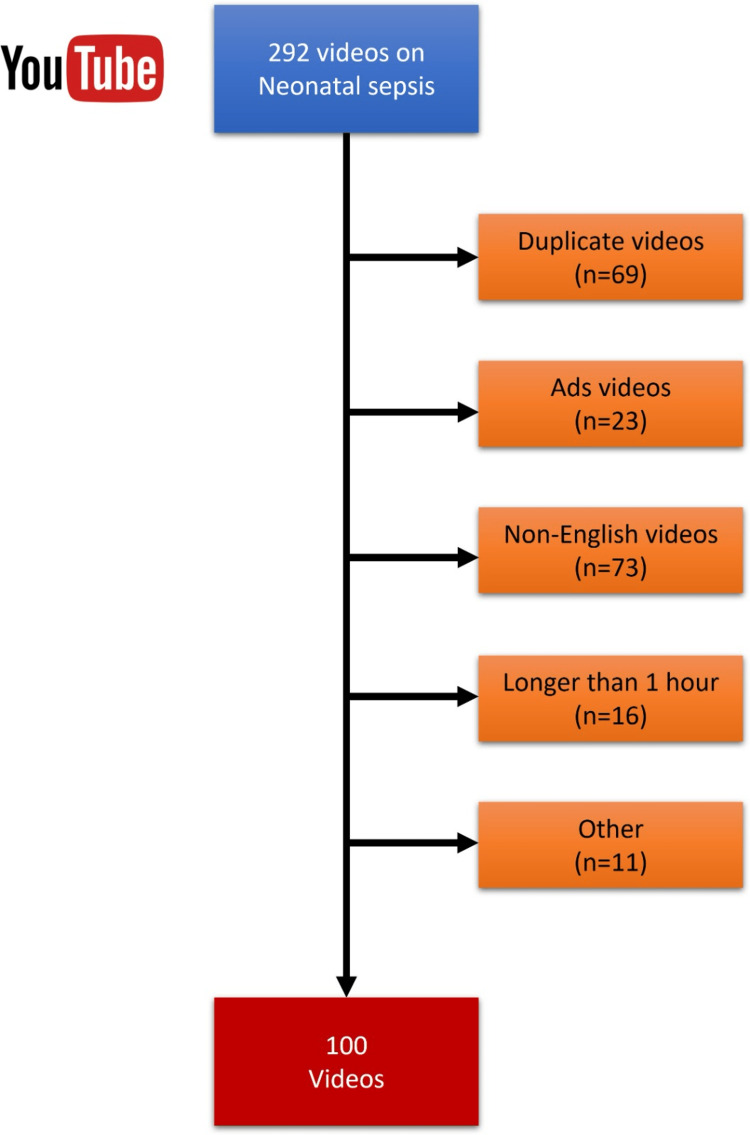
Flowchart of the reviewed videos

By consensus of the two researchers, the search term was determined as “neonatal sepsis”. The default filtering option “relevance” was used for the search. This search returned a total of 292 videos related to neonatal sepsis. Among these, duplicate videos, non-English videos, ads, videos longer than one hour and shorter than one minute, and others (without known creators) were excluded from analysis (Figure 1).

Finally, the remaining first 100 videos were subjected to analysis. Studies have shown that YouTube^TM^ users usually view the first results [13]. Videos’ basic characteristics were then recorded. These features included videos’ image type (real, presentation, animation), content (general information, treatment, diagnosis, education) and qualification of video creators (physicians, nurses, health channels, medical schools, TV shows, others). In addition, videos’ length, upload date, time since upload, comment and like counts were also recorded. The videos were assigned to two groups as the videos provided directly by physicians and those provided by non-physicians. The data obtained was compared between these groups.

Quality of the videos was measured using the Global Quality Scale (GQS) and reliability of the videos using the modified DISCERN (Quality Criteria for Consumer Health Information on Treatment Choices) tool. In addition, a third evaluation was carried out by the two observers together and the videos were divided into two classes as “useful” and “insufficient”.

Global Quality Scale

The Global Quality Scale was first used by Bernard et al. in 2007 to investigate the quality of health-related online information. The scale consists of five items scored on a five-point Likert scale. Ease of use, flow and quality of the information in the reviewed videos were scored between 1 (very poor) and excellent (5). The five items of GQS are shown in Table 1 [11].

**Table 1:** Global Quality Scale items

#	Item
1	Poor quality, poor flow of the site, most information missing, not at all useful for patients
2	Generally poor quality and poor flow, some information listed but many important topics missing, of very limited use to patients
3	Moderate quality, suboptimal flow, some important information is adequately discussed but others poorly discussed, somewhat useful for patients
4	Good quality and generally good flow, most of the relevant information is listed, but some topics not covered, useful for patients
5	Excellent quality and excellent flow, very useful for patients

DISCERN

The original DISCERN tool was developed in 1999 to evaluate written health-related information. This scale consisted of 16 items. The total score varied between 16 and 80 with high scores indicating increasing reliability of the information [14]. In 2021, Singh et al. modified the original DISCERN tool and adapted it to YouTube^TM^ videos with five items scored on the five-point Likert scale. A modified DISCERN score <3 shows poor reliability, a score of 3 indicates moderate reliability and >3 good reliability [11,15] (Table 2).

**Table 2:** Modified DISCERN scale items DISCERN: Quality Criteria for Consumer Health Information on Treatment Choices

#	Item
1	Are the aims clear and achieved?
2	Are reliable sources of information used?
3	Is the information presented balanced and unbiased?
4	Are additional sources of information listed for patient reference?
5	Are areas of uncertainty mentioned?

Statistical analysis

Data obtained in this study was statistically analyzed using the SPSS, version 25.0 (IBM Inc., Armonk, NY) statistical software. Normality of the variables was tested using the Kolmogorov-Smirnov method. An independent t-test was used to compare the continuous variables between the physician and non-physician videos on neonatal sepsis, while the chi-square test was used to analyze categorical data. Continuous variables are given as means±standard deviations and categorical variables as numbers and percentages. The agreement between the two researchers was evaluated with Cronbach alpha coefficients. The significance level was set at p<0.05.

## Results

**Figure 2:**
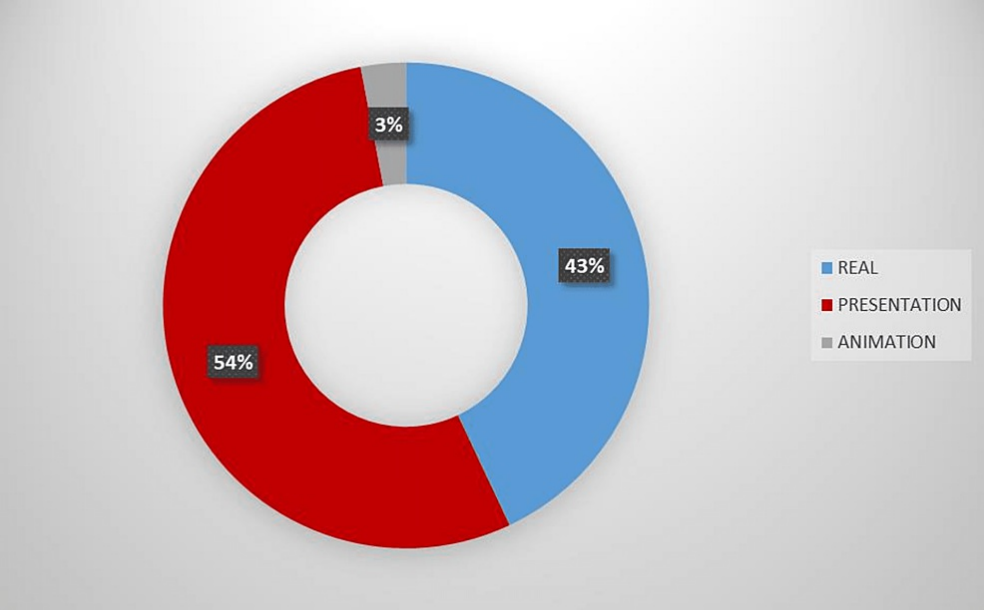
Image type of the reviewed videos

One hundred YouTube^TM^ videos pertaining to neonatal sepsis were included in the analysis. The total video length was 35.84 hours and total view count was 1,173,247. Image types of the videos are shown in Figure 2.

**Figure 3:**
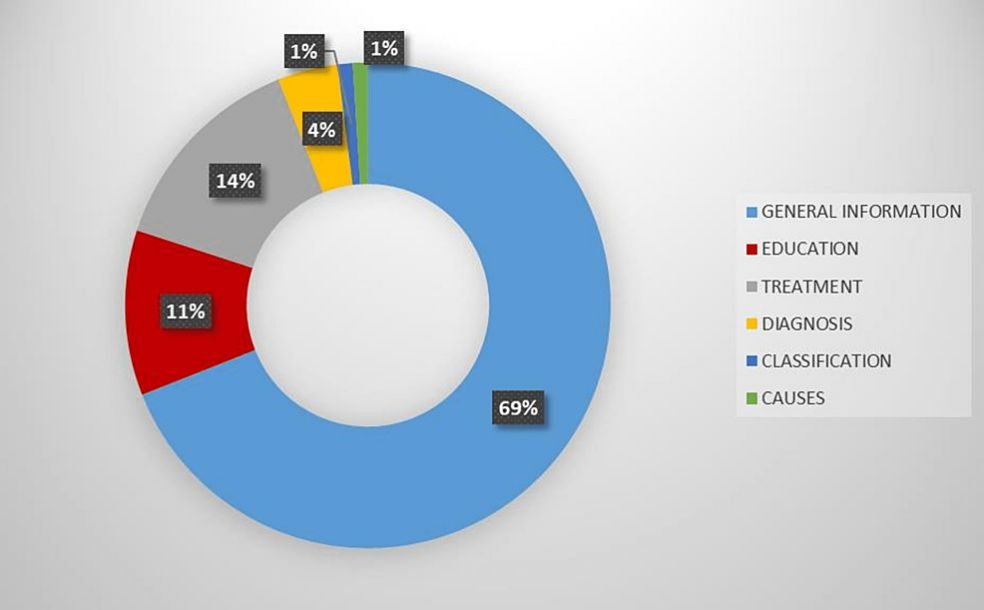
Distribution of video contents

The most common video content was general information about neonatal sepsis, education and diagnosis. The distribution of the video contents is presented in Figure 3.

**Figure 4:**
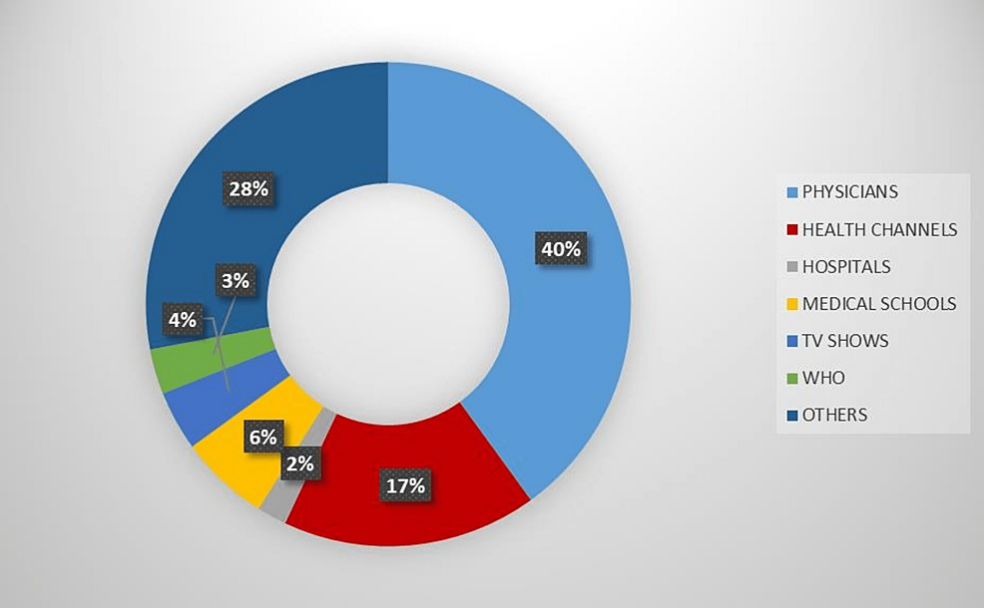
Distribution of the video creators

Qualification of the video creators was examined; the most common creators were physicians followed by other persons and health channels (Figure 4). General characteristics of all videos are given in Table 3.

**Table 3:** General characteristics of videos DISCERN: Quality Criteria for Consumer Health Information on Treatment Choices; GQS: Global Quality Scale

Parameter		
Total video length (hours)	35.84	
Total views (count)	1,173,247	
	Mean	±SD
Video length (min)	21.11	22.69
Views (count)	11850.98	23161.92
Time since upload (days)	1212.83	871.68
Daily views (count)	13.67	27.75
Comments (count)	11.29	25.81
Likes (count)	1743.53	16094.83
DISCERN	3.82	0.95
GQS	3.85	1.02

When the videos were subjectively evaluated on consensus of the two researchers, 65 (65%) videos were useful and 35 (35%) videos were insufficient.

The mean DISCERN score given by the first researcher was 3.72±0.96 and the mean DISCERN score given by the second researcher was 3.81±1.04. The mean GQS score given by the first researcher was 3.80±1.02 and the mean GQS score given by the second researcher was 3.83±1.08. An excellent agreement was found between the two observes in DISCERN and GQS values (Table 4).

**Table 4:** Agreement between the two researchers DISCERN: Quality Criteria for Consumer Health Information on Treatment Choices; GQS: Global Quality Scale

	Mean±SD	p	r	Cronbach α
DISCERN 1	3.72±0.96	p<0.01	0.867	0.888
DISCERN 2	3.81±1.04			
GQS 1	3.80±1.02	p<0.01	0.895	0.905
GQS 2	3.83±1.08			

The videos were divided into two groups according to the creators. Accordingly, 40 (40%) of videos were provided by professionals and 60 videos by non-professionals. There was a statistically significant difference between the physicians and non-physicians in terms of the mean DISCERN and GQS scores (Table 5). The other video characteristics did not show statistical significance between the physicians and non-physicians (Table 6).

**Table 5:** DISCERN and GQS scores of physicians and non-physicians DISCERN: Quality Criteria for Consumer Health Information on Treatment Choices; GQS: Global Quality Scale

	Physicians	Non-physicians	p
	Mean±SD		
DISCERN	4.56±0.50	3.32±0.85	p<0.01
GQS	4.66±0.47	3.30±0.91	p<0.01

**Table 6:** General characteristics of physicians’ and non-physicians’ videos

	Physicians	Non-physicians	p
	Mean±SD		
Video length (min)	24.47±21.49	18.83±23.38	0.537
Daily view	18.01±39.16	10.73±15.79	0.062
Comment	12.63±27.58	10.40±24.77	0.581
Like	159.20±230.17	2799.75±20779.67	0.110

According to the DISCERN scale results, reliability was found to be poor in 17 (17%) videos, moderate in 14 (14%) videos and good in 69 (69%) videos.

## Discussion

In the present study, we analyzed the reliability and quality and of the first 100 YouTube^TM^ videos related to neonatal sepsis. Both quality and reliability were significantly higher in the videos uploaded directly by physicians compared to those uploaded by other sources (health channels, TV channels, other persons, etc.). The mean video length varies highly among the studies according to the topic of interest. In the present study, the average video length was 21.11 minutes. In a study conducted by Aydin and Akyol investigating the quality of information available on YouTube^TM^ videos pertaining to thyroid cancer, the average video length was reported as 12.8 minutes [16]. In another study by Krakowiak et al., evaluating YouTube^TM^ as a source of patient information for meningiomas, the mean video length was 18.89 minutes [17]. Gonen et al. found the mean video length as 7.27 minutes [18]. As is seen, video length varies between the studies according to the subject of research. Our higher video length might be the result of the fact that a significant portion of the videos included educational information (including lectures) that lasted longer.

In our study, the oldest video content was uploaded on April 25, 2010, and the newest video content on July 02, 2022. The mean time since video upload was 1212.83 days. In a study by Kuru and Erken, the time since video uploading was reported as 2288.1 days [19]. Krakowiak et al. reported the time since uploading as 1688.71 days [17].

View counts is one of the parameters reflecting popularity of videos. In our study, the mean view count was 11850.98. The mean video views were found as 11,850.98. The mean video views were reported as 24,210.47 by Krakowiak et al. and 9292.5 by Gonen et al. [17,18]. Our mean video length fell in the range reported in previous studies.

The mean daily views eliminate time since uploading and is a more accurate measurement of view counts. The mean daily views were found as 13.67 in the present study. In a study by Cakmak and Mantoglu evaluating the quality and reliability of YouTube^TM^ videos related to pancreatic cancer, the average daily view was found as 279.77 [9]. In another study by Bai et al. assessing YouTube^TM^ videos as an information source for testicular torsion, the mean daily view was reported as 1150.62 [20]. As most other parameters, daily view count varies widely between the studies according the topic of research.

Comment and like counts reflect popularity of a video. In this study, the average comment count was 11.29 and the mean like count was 1743.53. In a study conducted by Kuru and Erken examining the quality and reliability of YouTube^TM^ videos pertaining to tears of the rotator cuff, the mean comment count was reported as 176.2 and the mean like count was 1811.2, similar to our like count [19]. In another study by Turhan and Ünsal evaluating the quality of YouTube^TM^ videos on hemorrhoidal disease, the average like count was reported as 3022.57 [21]. In another study by Yurdaisik analyzing the first 50 videos on YouTube^TM^ about breast cancer, the mean comment count was found as 535 and like count as 5,214 [22]. We think that the differences between the studies reflect the fact that comment and like counts vary according to public concerns and curiosity about the disease or topic of interest.

Quality and reliability depend on creators of the videos. In our study, 40% of the videos were provided by physicians, 17% by health channels, 6% by medical schools, 4% by TV shows, 3% by the affiliations of the WHO, 2% by hospitals and 28% by lay persons. We divided the creators as physicians and non-physicians and compared the variables between these two groups. No statistically significant difference was found between the two groups in terms of video length, daily view, comment and like counts (for all, p>0.05).

In a study by Yurdaisik, only 14% of the videos were uploaded directly by physicians, while the other creators included blog channels, news channels, patients, and health channels [22]. In the study by Kuru and Erken, 28% of the videos were provided by healthcare professionals and 72% by others [19]. Similarly, in a study conducted by Memioglu and Ozyasar analysing YouTube^TM^ contents as a source of information for myocarditis during the COVID‑19 pandemic, 28% of the videos were uploaded by physicians [23]. Although our rate of videos uploaded by physicians was higher compared to the other studies, more and more physicians are needed to upload high-quality and accurate video contents to guide patients correctly.

Reliability of the videos was determined with the modified DISCERN scale. In the present study, the mean DISCERN score was found as 4.56±0.50 for the videos uploaded directly by physicians and 3.32±0.85 for those uploaded by non-physicians (p<0.01). In the study by Memioglu and Ozyasar, the mean DISCERN score was found as 4.32±0.77 for the videos provided by professionals [23]. In a study by Onder and Zengin, the average DISCERN score was 4.0 for the videos provided by physicians and 2.0 for the videos provided by the independent users [6]. On the other hand, in a study by Sahin and Agar, the mean DISCERN score was found as 1.76±0.8 for the videos uploaded by physicians [24].

Quality of the videos was determined using the GQS score. In our study, the mean GQS score was found as 4.66±0.47 for the videos uploaded directly by physicians and 3.30±0.91 for those uploaded by non-physicians (p<0.01). In the study by Turhan and Ünsal, the mean GQS score was found as 3.49±1.023 in the videos provided by professionals [21]. In the study conducted by Memioglu and Ozyasar, the mean GQS score was found as 3.89±0.83 in the videos uploaded by physicians [23]. We think that our higher DISCERN and GQS scores in the videos uploaded by physicians depend on the fact that in our study, physicians presented information about a more specific disease such as neonatal sepsis in detail. In addition, there was an excellent agreement between the two researchers who reviewed the videos.

Study limitations

This study has several limitations. First, only English videos and the first 100 videos were included. However, as stated above, YouTube^TM^ users tend to view first results. Second, we made a snapshot assessment, whereas view, comment and like counts on YouTube^TM^ can change constantly. In addition, there may be potential biases, such as selection bias or the possibility that the 100 videos analyzed may not be representative of all videos related to neonatal sepsis. As a strength, to our knowledge, this study is the first in the literature to evaluate YouTube^TM^ videos pertaining to neonatal sepsis.

## Conclusions

According to the results of this study, both DISCERN and GQS scores were significantly higher for the videos uploaded directly by physicians. However, 17% of the videos were of poor reliability and could be misleading. Physicians should be encouraged to upload accurate informative videos about neonatal sepsis and direct parents to accurate sources of treatment. Also, parents and relatives of the patients should view videos created by professionals. Platforms such as YouTube^TM ^should develop a supervision process for healthcare-related videos and control them before they become available to public.
